# Proteomic Analysis of Marinesco–Sjogren Syndrome Fibroblasts Indicates Pro-Survival Metabolic Adaptation to SIL1 Loss

**DOI:** 10.3390/ijms222212449

**Published:** 2021-11-18

**Authors:** Francesca Potenza, Maria Concetta Cufaro, Linda Di Biase, Valeria Panella, Antonella Di Campli, Anna Giulia Ruggieri, Beatrice Dufrusine, Elena Restelli, Laura Pietrangelo, Feliciano Protasi, Damiana Pieragostino, Vincenzo De Laurenzi, Luca Federici, Roberto Chiesa, Michele Sallese

**Affiliations:** 1 Department of Innovative Technologies in Medicine and Dentistry, “G. d’Annunzio” University of Chieti-Pescara, 66100 Chieti, Italy; francesca.potenza@unich.it (F.P.); lindadibiase91@gmail.com (L.D.B.); annagiuliarug@gmail.com (A.G.R.); bdufrusine@unite.it (B.D.); damiana.pieragostino@unich.it (D.P.); delaurenzi@unich.it (V.D.L.); lfederici@unich.it (L.F.); 2 Center for Advanced Studies and Technology (CAST), “G. d’Annunzio” University of Chieti-Pescara, 66100 Chieti, Italy; maria.cufaro@unich.it (M.C.C.); a.dicampli@ibp.cnr.it (A.D.C.); laura.pietrangelo@unich.it (L.P.); feliciano.protasi@unich.it (F.P.); 3 Department of Pharmacy, “G. d’Annunzio” University of Chieti-Pescara, 66100 Chieti, Italy; 4 Department of Life, Health and Environmental Sciences, University of L’Aquila, 67100 L’Aquila, Italy; valeriapanella@alice.it; 5 Institute of Protein Biochemistry (IBP), Italian National Research Council (CNR), 80131 Napoli, Italy; 6 Department of Neuroscience, Istituto di Ricerche Farmacologiche Mario Negri IRCCS, 20156 Milano, Italy; elena.restelli@marionegri.it (E.R.); roberto.chiesa@marionegri.it (R.C.); 7 Department of Medicine and Aging Sciences, “G. d’Annunzio” University of Chieti-Pescara, 66100 Chieti, Italy

**Keywords:** protein folding, unfolded protein response, BiP, pathway analysis, fibroblast, neurodegenerative disease, autophagosome

## Abstract

Marinesco–Sjogren syndrome (MSS) is a rare multisystem pediatric disorder, caused by loss-of-function mutations in the gene encoding the endoplasmic reticulum cochaperone SIL1. SIL1 acts as a nucleotide exchange factor for BiP, which plays a central role in secretory protein folding. SIL1 mutant cells have reduced BiP-assisted protein folding, cannot fulfil their protein needs, and experience chronic activation of the unfolded protein response (UPR). Maladaptive UPR may explain the cerebellar and skeletal muscle degeneration responsible for the ataxia and muscle weakness typical of MSS. However, the cause of other more variable, clinical manifestations, such as mild to severe mental retardation, hypogonadism, short stature, and skeletal deformities, is less clear. To gain insights into the pathogenic mechanisms and/or adaptive responses to SIL1 loss, we carried out cell biological and proteomic investigations in skin fibroblasts derived from a young patient carrying the SIL1 R111X mutation. Despite fibroblasts not being overtly affected in MSS, we found morphological and biochemical changes indicative of UPR activation and altered cell metabolism. All the cell machineries involved in RNA splicing and translation were strongly downregulated, while protein degradation via lysosome-based structures was boosted, consistent with an attempt of the cell to reduce the workload of the endoplasmic reticulum and dispose of misfolded proteins. Cell metabolism was extensively affected as we observed a reduction in lipid synthesis, an increase in beta oxidation, and an enhancement of the tricarboxylic acid cycle, with upregulation of eight of its enzymes. Finally, the catabolic pathways of various amino acids, including valine, leucine, isoleucine, tryptophan, lysine, aspartate, and phenylalanine, were enhanced, while the biosynthetic pathways of arginine, serine, glycine, and cysteine were reduced. These results indicate that, in addition to UPR activation and increased protein degradation, MSS fibroblasts have profound metabolic alterations, which may help them cope with the absence of SIL1.

## 1. Introduction

Marinesco–Sjogren syndrome (MSS) is a rare multisystem disorder of infancy for which there is no cure [1]. MSS patients present with cerebellar ataxia, muscle weakness, and congenital/early cataracts, with this triad being the basis for clinical diagnosis. Other symptoms include intention tremor, mild to severe mental retardation, hypogonadism, short stature, and skeletal deformities [2,3].

Cerebellar degeneration is the main cause of poor motor coordination and speech difficulties, while myopathy with replacement of skeletal muscle fibers with fat tissue is responsible for the reduced strength [4]. A delayed migration of cortical neurons might contribute to the intellectual disability [5].

About 60% of MSS patients carry homozygous or compound heterozygous mutations in the *SIL1* gene that make the SIL1 protein unstable, eventually leading to its loss [2,6,7,8]. SIL1 is an ATP-exchange factor for the endoplasmic reticulum (ER) chaperone-binding immunoglobulin protein (BiP), which plays a central role in protein folding. BiP’s ability to bind unfolded proteins and release the folded substrate is tightly regulated by a cycle of ATP binding, hydrolysis, and nucleotide exchange. SIL1 binds to ADP-bound BiP to catalyze the release of ADP, allowing rebinding of ATP [9]. If this nucleotide exchange is defective, as in the case of SIL1 loss, BiP remains associated with its client protein, ultimately leading to the accumulation of unfolded proteins. This change in proteostasis activates the unfolded protein response (UPR) and triggers the formation of autophagic vacuoles [2,4,10].

The UPR is a complex signaling pathway whose purpose is to restore ER proteostasis by inhibiting protein synthesis, increasing the ER protein-folding capacity and degrading unfolded proteins. The three primary effectors of the UPR are the PRK-like endoplasmic reticulum kinase (PERK) and inositol-requiring enzyme 1 (IRE1), and the activating transcription factor 6 (ATF6) [11,12]. These are ER transmembrane proteins, which are kept inactive by binding of BiP to their sensor domains in the ER lumen. Upon accumulation of unfolded/misfolded proteins in the ER, BiP detaches, and they are activated. Activated PERK phosphorylates the alpha subunit of eukaryotic translation initiation factor 2 (eIF2α). This inhibits mRNA translation, reducing global protein synthesis. Some specific mRNA, however, are preferentially translated, including the activating transcription factor 4 (ATF4). Among the genes induced by ATF4, there are multiple autophagy-related genes. Autophagy is an important catabolic pathway that recovers energy by degrading and recycling organelles and proteins [13]. Activated IRE1 has endoribonuclease activity, which degrades cell mRNAs through a mechanism known as regulated IRE1-dependent decay (RIDD), contributing to reducing protein synthesis [12]. IRE1 also removes an intron from XBP-1 mRNA, generating a spliced mRNA, which encodes a more active form of the XBP-1 transcription factor (sXBP-1), which enhances the transcription of genes involved in protein folding (chaperones), secretion, and ER-associated degradation (ERAD). During ER stress, ATF6 moves from the ER to the Golgi, where it undergoes regulated intramembrane proteolysis, releasing a transcriptionally active N-terminal portion of 50 kDa, which enters the nucleus and promotes transcription of UPR genes, including XBP-1 and BiP [11,12]. If cells are unable to handle the unfolded protein load, protracted ATF4 synthesis may lead to apoptosis [12].

The mechanisms by which SIL1 mutations cause MSS are not yet fully understood, but loss of SIL1 protein with disruption of ER proteostasis and maladaptive UPR may contribute to the pathogenic process. We found that the PERK inhibitor GSK2606414 partially restores proteostasis, attenuates apoptosis in SIL1-deficient cells, and has beneficial effects in the woozy mouse model of MSS, attenuating PC degeneration and muscle pathology [14,15,16]. Another study showed that the chemical chaperone tauroursodeoxycholic acid (TUDCA) slightly attenuates ER stress-induced apoptosis in MSS patients’ lymphoblastoid cells [17].

SIL1 loss affects some specific tissues while others show no cytopathic signs although they do exhibit proteostatic changes [2]. For example, woozy mouse motor neurons show a characteristic change in their proteome and are more vulnerable to stressful conditions; however, they show no evidence of degeneration in the absence of specific stressors [5]. This tissue selectivity is even more striking within the cerebellum, where Purkinje cells are spared in lobules IX and X of the vestibulocerebellum while they degenerate in the remaining lobules [10]. The reasons for such selectivity of effects are not clear.

A recent study indicated that the protein signature of human skin fibroblasts may help understand the pathogenic mechanisms of neuromuscular diseases [18]. We decided to study the proteome of MSS patient fibroblasts (HF-MSS) with a view to understanding the molecular changes involved in the pathogenesis of this syndrome.

We provide evidence that MSS fibroblasts present signs of UPR and proliferation of autophagic vacuoles. In line with UPR-inhibited protein synthesis, the spliceosome and ribosome were downregulated. We also observed a profound reorganization of the cellular metabolism, which uses lipids and amino acids for energy purposes, producing ATP through oxidative phosphorylation. These molecular alterations resulted in a strong inhibition of cell proliferation. Thus, HF-MSS activate adaptive responses that allow them to survive despite reduced ER folding capacity.

## 2. Results

### 2.1. The Cellular Hallmarks of MSS Are Recapitulated in Cultured Skin Fibroblasts

**Figure 1:**
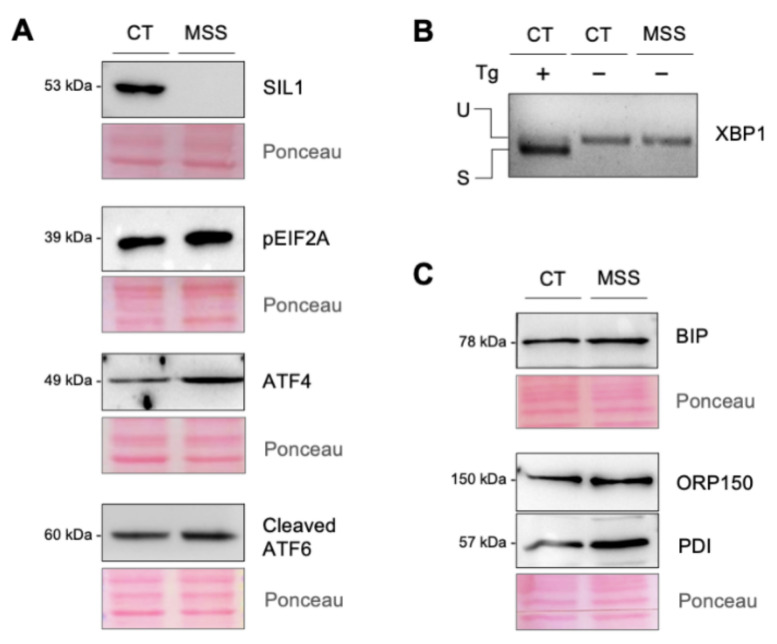
UPR activation in MSS patient fibroblasts. (**A**,**C**) HF-CT (CT) and HF-MSS (MSS) cultured under standard growth conditions were analyzed by Western blotting to assess the expression of SIL1, ATF4, ATF6, and the phosphorylated form of EIF2α (**A**), BiP, ORP150, and PDI (**C**) as indicated. Immunoblots are representative of at least two independent experiments. Ponceau S staining of the blots is shown to control for equal protein loading. (**B**) XBP1 splicing was assessed by PCR. HF-CT (first lane) treated with 1 μM thapsigargin (Tg) for 6 h was used as a positive control. The higher and lower bands represent the unspliced (U) and spliced (S) forms of XBP1.

We examined whether HF-MSS displayed molecular and morphological changes that have been reported in other cell types lacking SIL1, including UPR activation and increased autophagy [2,3]. Western blot analysis confirmed the absence of SIL1 protein, and showed a modest activation of the PERK and ATF6 branches of the UPR in HF-MSS cells. Specifically, the levels of ATF4 and eIF2-α phosphorylated at threonine 52, which are readouts of PERK activation, were increased in comparison to control juvenile skin fibroblasts (HF-CT) (Figure 1A). There was also an increase in the cleaved form of ATF6, indicating stress-activated processing of this sensor (Figure 1A). There was no increase in sXBP1, indicating no IRE1 activation (Figure 1B). We also examined the expression of a few chaperones that are induced during ER stress. BiP and protein disulfide isomerase (PDI) were upregulated in HF-MSS in comparison to HF-CT, while oxygen-regulated protein of 150kD (ORP150) was unaffected (Figure 1C).

**Figure 2:**
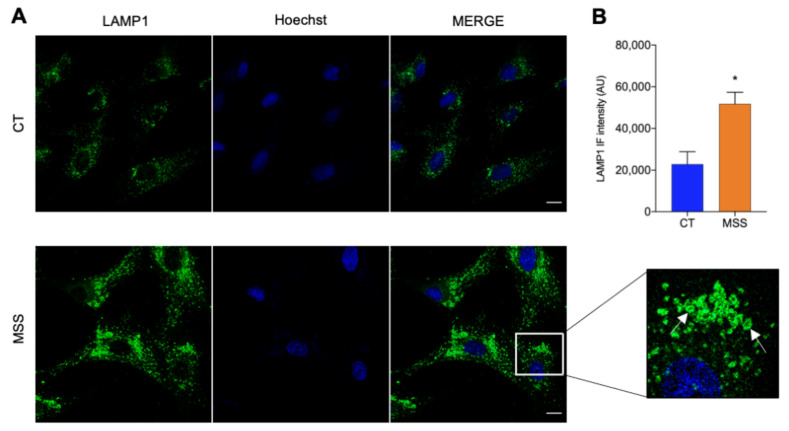
LAMP1-positive structures increase in HF-MSS cells. (**A**) HF-CT (CT) and HF-MSS (MSS) were plated on glass coverslips, fixed in PFA 4%, and processed for immunofluorescence. Confocal images of cells immunostained for LAMP1 (green) and reacted with Hoechst 33342 (blue) to stain the nuclei. The merge of green and blue channels is represented in the third panel. A magnification of the squared area is also shown. White arrows indicate ring-like structures. Scale bar: 20 µm. (**B**) Quantification of total LAMP1 staining shown in A. * *p* < 0.05.

Autophagic vacuoles are a consistent feature of the cellular pathology of MSS, seen in the skeletal muscle of patients, in the muscle and PCs of woozy mice, and in several cell lines in which SIL1 expression is genetically reduced. To investigate the presence of such structures in HF-MSS, cells were grown on cover slips, fixed, and labeled with an anti-lysosomal-associated membrane protein 1 (LAMP1) antibody. Fluorescence microscopy analysis showed an impressive number of intensely labeled ring-like structures, most likely enlarged lysosomes or autophagic vacuoles (Figure 2A). In control cells, these structures were rarely visible, with cells showing the expected cytosolic LAMP1-immunopositive dots (Figure 2A,B).

**Figure 3:**
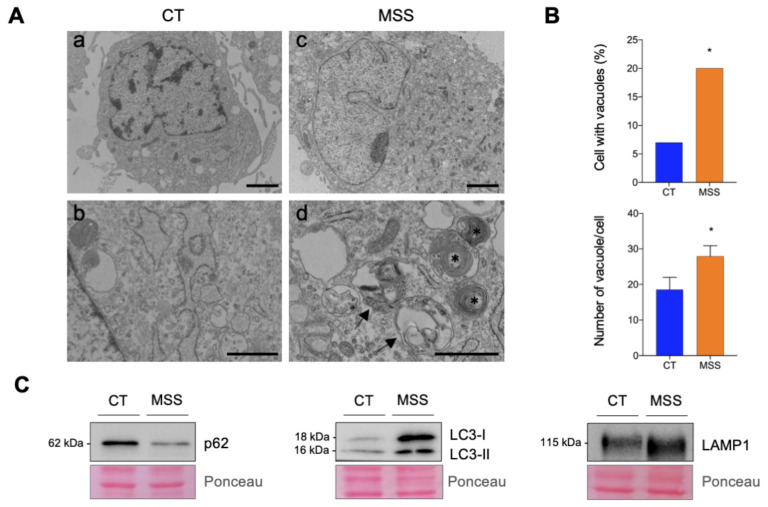
Electron microscopy (EM) and immunoblot analysis of autophagic vacuoles. (**A**) Ultrastructural alterations in HF-MSS. Representative EM micrographs of HF-CT (a) and HF-MSS (c) cells. Details at higher magnification of the cytoplasm of HF-CT (b) and HF-MSS (d) cells. HF-MSS cells accumulate vacuoles containing large clusters of membranes (arrowheads) and multilamellar bodies (asterisks). Scale bars: a and c: 2 μm; b and d: 1 μm. (**B**) Upper graph, percentage of HF-CT and HF-MSS cells exhibiting vacuoles/multilamellar bodies (*n* = 100 cells/group); lower graph, average number of vacuoles/multilamellar bodies per cell calculated by considering only the cells showing such structures. Statistical significance Chi square test (lower) and *t*-test (upper) * *p* < 0.05. (**C**) HF-CT (CT) and HF-MSS (MSS) cultured under standard growth conditions were analyzed by Western blotting for the expression of p62/SQSTM1 (p62), LC3, and LAMP1. Immunoblots are representative of at least two independent experiments. Ponceau S staining of the blots is shown to control for equal protein loading.

Electron microscopy (EM) examination detected a higher percentage of HF-MSS cells exhibiting cytoplasmic accumulation of vacuoles containing large clusters of membranes and multilamellar bodies than in HF-CT (Figure 3A, compare panels b and d; and Figure 3B). These structures were also more numerous in HF-MSS than in HF-CT cells (Figure 3B).

Finally, Western blot analysis detected an upregulation of LC3-I, LC3-II, and LAMP1, and a downregulation of sequestosome1 (SQSTM1/p62) (Figure 3C). The increased amount of lipidated LC3-II suggests either increased autophagosome formation or impaired autophagic degradation. SQSTM1/p62 acts as a receptor for ubiquitinated proteins targeted for degradation and can be itself degraded by autophagic vacuoles. Taken together, these data suggest an enhancement of the autophagic degradation pathway.

Cell viability and the amount of apoptotic cell death, monitored by trypan blue staining and caspase-3 activation, were comparable in HF-MSS and control cells (Supplementary Materials Figure S1A,B).

This initial characterization indicated that, consistent with what has been observed in other MSS cell models, HF-MSS experience ER stress with UPR activation [16], but this does not result in reduced cell survival or apoptotic cell death.

### 2.2. Proteomics of HF-MSS Identifies 625 Differentially Expressed Proteins

We used a proteomic approach to identify changes in HF-MSS proteostasis, which may provide insights into the molecular pathogenesis of MSS and/or adaptive responses to SIL1 loss. Patient and control fibroblasts were analyzed by shotgun proteomics as described in the Materials and Methods.

**Figure 4:**
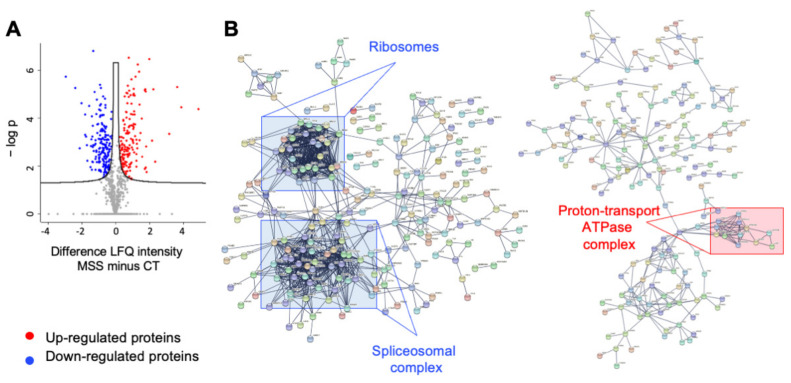
Proteomic analysis graphical networks. (**A**) Volcano Plot: Statistical analysis of differentially expressed proteins between HF-CT and HF-MSS cells, run by Perseus. LQF intensity of HF-MSS minus that of HF-CT was plotted against the negative logarithm of the *p*-values. (**B**) STRING network of proteins downregulated (left) and upregulated (right) in HF-MSS, confidence level set at 0.9. Squares indicate examples of clusters of interacting proteins involved in the indicated functions.

Raw data were processed using MaxQuant and the peak lists searched using the Andromeda peptide search engine against the UniProt database supplemented with frequently observed contaminants and containing forward and reverse sequences. Normalization and statistical analysis were carried out with Perseus, setting the false discovery rate (FDR) to 1%. Univariate statistical analysis was used to define the significantly differential proteins between control and patient fibroblasts (*p*-value < 0.05). Results are illustrated in a Volcano plot (Figure 4A). Pearson correlation of protein LFQ intensities between HF-MSS and HF-CT showed a high R^2^ value (0.92), indicating highly reproducible, relative label-free quantification in the two biological conditions studied. The highest protein density interpolates the mainline, consistent with a high degree of reproducibility and the absence of artefacts in cell culture (Supplementary Materials Figure S2).

We identified 625 differentially expressed (DE) proteins, of which 305 (49%) were significantly downregulated and 320 (51%) were significantly upregulated (Supplementary Materials Table S1).

### 2.3. Ontology Analysis of Differentially Expressed Proteins

The DE proteins were examined through STRING (https://string-db.org/ (accessed on 1 July 2021)), Gene Ontology (GO) enrichment analysis, and Ingenuity Pathway Analysis (IPA, Qiagen, Hilden, Germany). STRING, a protein–protein interaction (PPI) database (default settings: medium confidence), identified a network of 625 nodes with an average of 26 interactions each (average node degree 25.8). The expected number of edges by chance was estimated to be 3989, while our network presented 8031 edges and a PPI enrichment *p*-value < 1.0 × 10^−16^, indicating that the identified proteins belong to functional pathways affected in HF-MSS. By enhancing the confidence levels of the PPI to the highest level (0.9), the number of edges decreased to 2534 and the node degree average to 8.11, but the PPI enrichment remained highly significant (*p* < 1.0 × 10^−16^), underscoring the quality of the proteomic data. Subnetworks of downregulated and upregulated proteins are shown in Figure 4B and Figure 4C, respectively.

GO enrichment analysis provided by STRING showed several enriched “Biological Processes” including mRNA metabolic process (83 proteins, *p* < 2.5 × 10^−21^), mRNA splicing via spliceosome (45 proteins, *p* < 6.16 × 10^−15^), translation initiation (30 proteins, *p* < 1.17 × 10^−12^), and SRP-dependent co-translational protein targeting to membrane (23 proteins, *p* < 6.11 × 10^−11^). Proteins belonging to these processes were generally downregulated, indicating a reduction of mRNA maturation and translation. Proteins belonging to vesicle-mediated transport (139 proteins, *p* < 6.02 × 10^−21^), oxidation-reduction (94 proteins, *p* < 4.04 × 10^−19^), and small molecule metabolic processes (134 proteins, *p* < 1.21 × 10^−17^) were also enriched, although the direction of the changes depended on the specific process under consideration (see Table 1 and Table S2).

Amongst the “Molecular Functions” enriched in the DE proteins is worth mentioning RNA binding (86 proteins, *p* < 2.57 × 10^−17^), oxidoreductase activity (75 proteins, *p* < 8.7 × 10^−16^), nucleotide binding (134 proteins, *p* < 2.45 × 10^−12^), cytoskeletal protein binding (71 proteins, *p* < 4.82 × 10^−10^), proton-transporting ATPase activity, rotational mechanism (9 proteins, *p* < 4.97 × 10^−6^), integrin binding (19 proteins, *p* < 2.30 × 10^−6^), and cell adhesion molecule binding (24 proteins, *p* < 3.68 × 10^−6^). Except for proteins belonging to the RNA and cytoskeletal protein binding functions, which were generally downregulated, the other molecular functions included a preponderance of upregulated proteins. However, the presence of numerous proteins moving in the opposite direction makes it difficult to draw any firm conclusion on the consequences of these alterations for the cell (see Table 2 and Table S3).

The “Cellular Components” enriched in the DE proteins include organelles (566 proteins, 1.37 × 10^−50^), endoplasmic reticulum (120 proteins, *p* < 9.40 × 10^−13^), vesicle (173 proteins, *p* < 4.41 × 10^−24^), melanosome (35 proteins, *p* < 1.55 × 10^−20^), ribonucleoprotein complex (89 proteins, *p* < 2.33 × 10^−22^), vacuole (74 proteins, *p* < 4.05 × 10^−17^), lysosome (67 proteins, *p* < 1.24 × 10^−16^), and actin cytoskeleton (42 proteins, *p* < 1.75 × 10^−8^). Organelle proteins were either up- or downregulated, with those belonging to the melanosomes, vacuoles, lysosomes, and endoplasmic reticulum being mainly upregulated, and those belonging to the actin cytoskeleton and ribonucleoprotein complex mainly downregulated. A more extensive list of cellular components is reported in Table 3 and Table S4.

This analysis suggests that loss of SIL1 reduces global protein synthesis and potentiates the organelles deputed to protein degradation, as expected during UPR. However, in many cases, we could not draw firm conclusions because the very same protein can participate in multiple pathways with specific outcomes. Thus, to shed light on the cell machinery/functions involved in MSS, we sought for altered pathways using the KEGG database.

### 2.4. Analysis of Functional Pathway by KEGG

Pathway enrichment analysis, based on KEGG, revealed the involvement of several altered pathways, including spliceosome, RNA transport, and ribosomes whose proteins were downregulated (Table 4). This may be interpreted as a depotentiation of the machineries devoted to mRNA maturation, transport, and translation to reduce protein synthesis during UPR.

Proteins belonging to phagosome and lysosome pathways were also enriched but upregulated (Table 4 and Table S5). Generally, newly synthesized proteins that remain unfolded in the ER are degraded via the proteosome and/or the phagolysosome pathways [12,19,20]. Thus, these results suggest that phagolysosome is the preponderant degradation pathway activated in HF-MSS cells. This is in line with previous reports showing a proliferation of lysosome-based degradative organelles in MSS [4,14,16,21]. Finally, we observed an enrichment of metabolic pathways including fatty acid metabolism, tricarboxylic acid (TCA) cycle, glycolysis/gluconeogenesis, pentose phosphate, and amino acid degradation/biosynthesis (Table 4 and Table S5).

A more detailed analysis of these pathways indicated a downregulation of the fatty acid synthase and an upregulation of enzymes involved in fatty acid catabolism (beta oxidation), suggesting an increased need for acetyl-coenzyme A, the final product of this metabolic pathway. Acetyl-coenzyme A can be conveyed into the TCA cycle for energy production or used as a biosynthetic precursor for sterol synthesis. Interestingly, all the enzymes in the TCA cycle were upregulated, suggesting that most acetyl-coenzyme A follows this pathway, perhaps because these cells need large amounts of ATP (Table 4). In line with this, ATP5F1B and ATP5MG, two subunits of the mitochondrial ATP synthase, were upregulated (Supplementary Materials Table S1). Some cholesterol biosynthetic enzymes were also upregulated (Supplementary Materials Table S6), possibly reflecting an increased need of cholesterol to build up the membranes accumulating in the vacuoles and multilamellar bodies (Figure 3A,B) [22].

The glycolysis/gluconeogenesis and pentose phosphate pathways were enriched although the available data do not clarify whether these pathways were activated or inhibited, as key glycolytic enzymes, including phosphofructokinase (PFKL), dihydrolipoamide dehydrogenase (DLD), enolase (ENO1), and triosephosphate isomerase (TPI1), were either upregulated or downregulated (Supplementary Materials Table S1). Likewise, in the pentose phosphate pathway, the hexose-6-phosphate dehydrogenase (H6PD) was upregulated, while transketolase (TKT) expression was reduced.

Remarkably, various enzymes belonging to the amino acid degradation pathways, including the catabolism of valine, leucine, isoleucine, and tryptophan, were upregulated (Table 4 and Table 5). The enzymes aldehyde dehydrogenase 3 and 7 (ALDH3A2, ALDH7A1), and malate dehydrogenase 2 (MDH2), involved in the degradation of lysine, aspartate, and phenylalanine, were also increased (Supplementary Materials Table S1). Furthermore, we observed a downregulation of the arginine, serine, glycine, and cysteine biosynthetic pathways (Table 1, Table 4 and Table 5). This metabolic rewiring suggests that, with the protein synthesis rate reduced, as shown by PERK activation (Figure 1A; see also below), the amino acids supply exceeds the cell needs and the surplus is degraded and conveyed into the TCA cycle for energetic purposes.

Interestingly, several lysosomal enzymes involved in the glycan and glycosaminoglycan degradation pathways were upregulated. This could reflect the cell’s need to recycle unfolded proteins as an alternative energy source.

### 2.5. Analysis of Functional Pathway by IPA

To obtain a broader picture of the pathways possibly affected by SIL1 loss, data were further investigated using Ingenuity Pathway Analysis (IPA), a state of the art omics data mining software. IPA identified 170 significantly affected “Canonical Pathways” (Table 5 and Table S6). This large number of disrupted pathways is in line with previous studies, which showed that proteins with altered expression belong to all cellular compartments [23]. Among the affected canonical pathways, IPA confirmed phagosome, beta oxidation of fatty acids, TCA cycle, and degradation of amino acids in accordance with KEGG enrichment analysis (Table 4). Furthermore, it is worth mentioning the identification of mitochondrial dysfunction and inhibition of eIF2 signaling (Table 5), which is in agreement with the activation of the PERK pathway (Figure 1A). The caveolae-mediated endocytosis signaling also appeared to be affected (Table 5). The complete list of affected canonical pathways is shown in Supplementary Materials Table S6.

Finally, we categorized the “Disease or Functions” that are predicted to be increasing or decreasing on the basis of the DE proteins. IPA predicted that HF-MSS have increased phagocytosis, fatty acid metabolism, and cell death, in line with the above canonical pathways (Table 6 and Table S7). The DNA repair mechanisms and recombination were predicted to be decreased (Table 6). The cell cycle progression was also decreased, although its z-score, a statistical parameter that indicates whether a disease or function is up or downregulated, did not reach the threshold of significance to confirm a decrement (Table 6 and Table S7). Surprisingly, IPA indicates a decrease for movement disorders, motor dysfunction, and neurodegeneration (Table 6 and Table S7). However, a thorough examination of the proteins underlying this outcome revealed that these were mainly lysosomal enzymes involved in the breakdown of glycosphingolipids and glycosaminoglycans. These enzymes are generally downregulated or inactive in lysosomal storage disorders and gangliosidosis and lead to neurodegeneration [24]; therefore, their upregulation in HF-MSS led to the inappropriate categorization of decreased neurodegeneration and movement disorders.

### 2.6. Validation of the Proteomics

**Figure 5:**
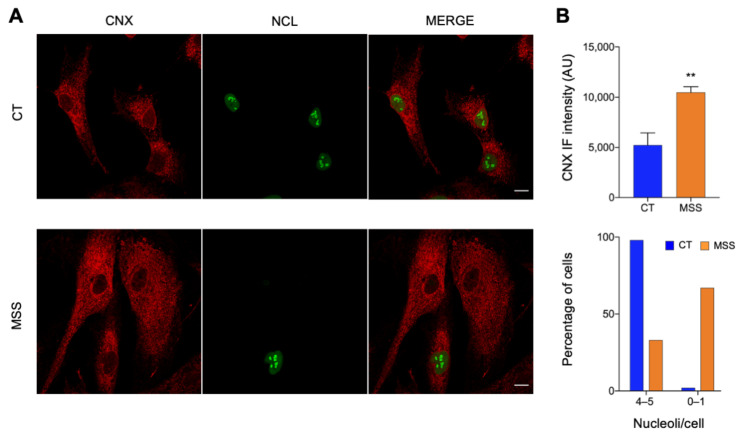
Fluorescence microscopy of representative DE proteins. (**A**) HF-CT (CT) and HF-MSS (MSS) were plated on glass coverslips, fixed in PFA 4%, and processed for immunofluorescence. Confocal images of cells stained for CNX (red) and NCL (green) are shown. The merge of green and red channels is shown in the third panel. Scale bar: 20 µm. (**B**) Quantification of total CNX staining and the number of NCL-positive nucleoli/cell of the experiment shown in A. Blue and red bars indicate HF-CT and HF-MSS, respectively. ** *p* < 0.01.

**Figure 6:**
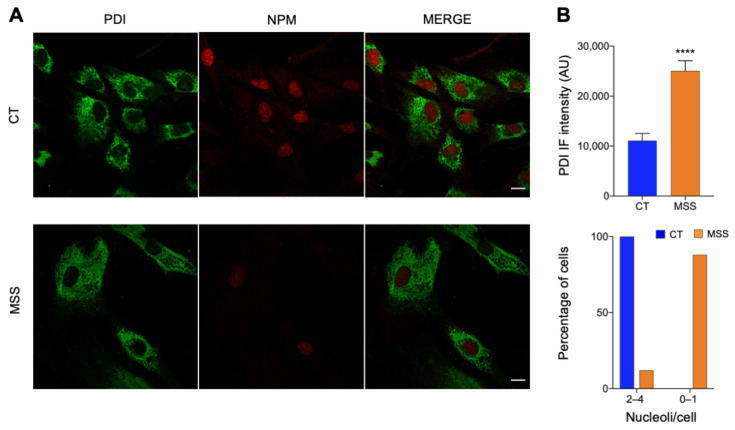
Fluorescence microscopy validation of proteomic analysis. (**A**) HF-CT (CT) and HF-MSS (MSS) were plated on glass coverslips, fixed in PFA 4%, and processed for immunofluorescence. Confocal images of cells stained for PDI (green) and NPM (red) are shown. The merge of green and red signal is also shown. Scale bar: 20 µm. (**B**) Quantification of total PDI staining and the number of NPM-positive nucleoli/cell of the experiment shown in A. Blue and red bars indicate HF-CT and HF-MSS, respectively. **** *p* < 0.0001.

The main proteomic results were validated by analyzing the expression of selected proteins by immunofluorescence and Western blot. The pathway analysis indicated a marked downregulation of the machineries responsible for the processing and translation of RNA. To corroborate this finding, we monitored the expression of nucleolin (NCL) and nucleophosmin (NPM), two nucleolar proteins involved in the biogenesis of ribosomes. Both were clearly visible in the nuclei of HF-CT while they were absent or barely detectable in many HF-MSS cells, with only a minority of cells showing normal levels of NCL and NPM in the nucleus (Figure 5 and Figure 6).

Proteomics also identified upregulated chaperones and lysosomal proteins, including calnexin, PDI, and LAMP1. Immunofluorescence analysis of HF-MSS showed misshaped and enlarged ER cisternae containing high quantities of calnexin and PDI (Figure 5 and Figure 6). Notably, approximately 30% of the HF-MSS cells looked larger than normal. This was confirmed by quantitative analysis: on average, HF-MSS were 37% larger than HF-CT fibroblasts, with some cells exceeding the mean size of controls by nearly 400%. The larger cells showed the most striking abnormalities, such as poor nuclear staining for NCL and NPM, and larger amounts of PDI, suggesting that the enlarged cell size represents an aspect of the pathological phenotype (Figure 5 and Figure 6).

**Figure 7:**
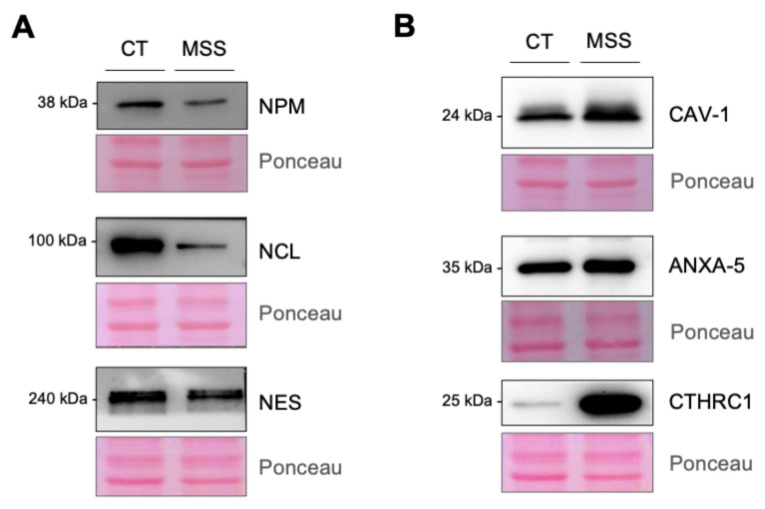
Western blot validation of proteomic analysis. (**A**,**B**) HF-CT (CT) and HF-MSS (MSS) cultured under standard growth conditions were analyzed by Western blotting to assess the expression of NPM, NCL, NES, CAV-1, ANXA-5, and CTHRC1 as indicated. The proteins downregulated or upregulated in HF-MSS are shown in A and B, respectively. Immunoblots are representative of at least two independent experiments. Ponceau S staining of the blots is shown to control for equal protein loading.

Western blotting of HF-MSS cell lysate confirmed the strong reduction of NCL, NPM, and nestin (NES) levels detected by proteomics (Figure 7A). The expression of caveolin-1 (CAV-1), annexin-V (ANXA-5), and collagen triple helix repeat containing 1 (CTHRC1) was upregulated, confirming the proteomics data (Figure 7B).

In conclusion, complementary approaches confirmed the changes in protein expression found by proteomics.

Finally, based on strong downregulation of ribosome and RNA splicing machinery, and on IPA “Disease or Functions” inferences (Table 6), we decided to experimentally evaluate the proliferation rate of HF-MSS cells. Cell growth, examined by the incorporation of a thymidine analogue (EdU), was strongly reduced in comparison to control cells (Supplementary Materials Figure S3).

## 3. Discussion

Loss of function of the BiP cochaperone SIL1 in MSS causes dramatic cerebellar and skeletal muscle degeneration, variable abnormalities in other tissues such as the eyes and bones, but no major defects in most organs, despite SIL1 being expressed in all body cells [2,3]. ORP150 can act as a nucleotide exchange factor for BiP, and slightly higher basal or induced levels of ORP150 have been proposed to compensate SIL1 loss in surviving cells [25]. In the present study, we examined global proteome changes in fibroblasts isolated from an MSS patient, with the aim of uncovering how unaffected MSS cells cope with the absence of SIL1. We found no increase in ORP150 compared to control fibroblasts but higher levels of ER stress markers, with downregulation of protein synthesis and upregulation of degradative processes, consistent with activation of the unfolded protein response. In addition, we detected protein changes indicative of a profound rewiring of signaling and metabolic circuitries, which may contribute to cell survival in the absence of SIL1.

Initial characterization of HF-MSS indicated the presence of a mild UPR based on increased eIF2α phosphorylation and ATF6 cleavage. Endo-lysosome markers were also upregulated in line with the rise in the number of these organelles. The presence of this pathological phenotype is surprising because to date, no connective tissue-related problems have been reported in MSS. However, this initial analysis suggests that clinically unaffected cells recall the characteristic molecular pathology previously observed in MSS-affected cells [2,3].

Driven by these interesting results, we performed a proteomic analysis on HF-MSS cells. Comparison with control fibroblasts detected altered expression of more than 600 proteins. To shed light on these differentially expressed proteins, we performed ontology and pathway analysis using multiple web platforms. In HF-MSS cells, there was a clear downregulation of the machineries deputed to RNA splicing and transport. The ribosomes and RNA translation initiation were also downregulated. Some of these changes could be ascribed to a general reduction of protein synthesis related to UPR activation, although the downregulation of spliceosome was unexpected. Remarkably, a similar alteration has been reported in more than 300 cancer cell lines exposed to a number of different anticancer treatments, and it is considered a stress response mechanism that promotes survival of cancer cells [26]. The spliceosome is downregulated during fibroblast senescence [27]. In line with accelerated cell aging, HF-MSS showed a downregulation of histone proteins, a well-recognized sign of senescence [28]. Importantly, one of the key features of senescent cells is their increased size [29]. These results, and the fact that the HF-MSS proliferation rate was reduced, suggest that these cells achieve a sort of senescent state in order to survive.

We also found that HF-MSS cells have profound metabolic changes. They show an increase in catabolism of fatty acids and amino acids released from lysosomal protein degradation; the TCA cycle is potentiated to handle the acetyl-CoA produced by a boosted beta oxidation and catabolized amino acid skeletons, and mitochondrial ATP synthase is upregulated, suggesting increased production of ATP through oxidative phosphorylation. A similar response can be triggered by downregulation of histones in yeast cells [30]. On the other hand, metabolites of the TCA cycle control multiple physio-pathological cellular functions, including gene expression via histone methylation and acetylation [31].

The ER consumes high amounts of ATP for protein folding, trafficking, and maintenance of ion homeostasis but does not possess its own ATP generation system [32]. The UPR, challenging these basic ER functions, increases the usage of ATP in this organelle. Interestingly, the ATP used in the ER at steady state is preferentially generated by mitochondria via oxidative phosphorylation [33]. The dependence of ER from mitochondrial ATP further increases during the UPR. ATP enters the ER through the solute carrier family 35 member B1 (SLC35B1) present at the ER–mitochondria contact sites [32]. The ER–mitochondria contacts are considered important structures to maintain the coupling between ATP consumption and production rate, and we found that the expression of mitofusin-2, a structural protein of this contact site [34], is markedly increased in HF-MSS (data not shown).

Overall, our data indicated that HF-MSS cells undergo metabolic changes to support the increased need of ATP in the ER to sustain a challenged ion homeostasis and folding activity.

## 4. Materials and Methods

### 4.1. Reagents

The following antibodies were used for WB and/or IF as indicated: SIL1 (Proteintech #24110-1-AP); ATF4 (Cell Signaling #11815); ATF6B (Proteintech #15794-1-AP); EIF2α (Santa Cruz #sc-11386); pho-EIf2α (Cell Signaling #3597s); PDI (Assay Design #SPA-891); BiP (BD #610979); ORP150 (Abcam #EP5891); LC3B (Cell Signaling #2775); NCL (Cell Signaling #87792S); NPM (Cell Signaling #3542S); CTHRC1 (Abcam #ab85739); Anti SQSTM1 (BD Bioscience #610833); LAMP1 (Abcam #ab25630); CNX (Cell Signaling #2679S); NES (Millipore #AB5922); CAV-1 (Cell Signaling #3238); and GAPDH (Santa Cruz #sc-32233). Hoechst #33342 from Life Technology was used to counterstain nuclei. For PCR amplification of the spliced and unspliced form of human XBP1, the following primers were used: FW 5′-AGCTCAGACTGCCAGAGATCG-3′; REV 5′-AATCCATGGGGAGATGTTCTA-3′ [35]. Powders, buffers, and other commonly used reagents were supplied by Sigma unless otherwise noted and were of analytical grade or higher.

### 4.2. Cell Culture

Primary Human Dermal Fibroblast (NDHF Promo Cell #FB60C12350) supplied by Carlo Erba were used as the control cell line and compared with primary dermal fibroblast from a young Marinesco–Sjögren syndrome patient, supplied by Telethon Network of Genetic Biobanks—TNGB [36]. Cells were cultured in Dulbecco’s modified Eagle’s medium + GlutaMAX (GIBCO, 61965-026), supplemented with 10% of Fetal Bovine Serum (FBS) and 1% penicillin/streptomycin PenStrep (GIBCO, 15070-063). Cells were maintained at 37 °C, 5% CO_2_, and detached by Trypsin-EDTA 0.5% (GIBCO, 15400-054).

### 4.3. Fluorescence Immunostaining and Confocal Microscopy

Cells were plated on cover slips to 70% density and the day after fixed in 4% paraformaldehyde (Electron Microscopy Science) for 10 min at room temperature. Cells were subsequently incubated for non-specific site blocking and permeabilization in blocking solution [0.05% saponin, 0.5% bovine serum albumin, and 50 mM NH_4_Cl in PBS] for 30 min at room temperature. Primary antibody incubation was performed overnight at +4 °C. The day after, fluorescent Alexa Fluor-conjugated anti-IgG secondary antibody was incubated for 40 min at room temperature together with Hoechst, in order to counterstain nuclei. Signal was than detected with a confocal microscope (Zeiss LSM 800) and images were post-processed and analyzed by Zen 2.3 software (Zeiss, Jena, Germany). The total immunofluorescence intensity of the signals was measured by ImageJ software (National Institutes of Health, Bethesda, MD, USA). The total immunofluorescence intensity was then normalized for cell number. All experiments were carried out at least two times. The results are shown as arbitrary units (AU).

### 4.4. Western Blot Analysis and PCR

Cells were lysed in RIPA buffer [50 mM tris HCl (pH 7.6), 140 mM NaCl, 5 mM EDTA, 0.1% SDS, 1% NP40, 100 mM NaF, 0.1% Sodium deoxycholate] supplemented with protease (cOmpleteTM Tablets, Roche) and phosphatase (PhosSTOP tablets, Roche) inhibitors. Protein concentration was assessed by Bradford protein assay (Bio-Rad). Equal amounts of proteins were than resuspended in 4x Laemmli buffer [15% b-mercaptoethanol, 0.25 M Tris-HCl pH 6.8, 40% Glycerol, 8% SDS, 0.02% Blu Bromophenol], separated by SDS-PAGE, and transferred onto nitrocellulose membrane. The presence of proteins on the nitrocellulose membranes and their quantity was assessed by Ponceau S staining [0.5% (*w*/*v*) Ponceau S dissolved in 1% (*v*/*v*) acetic acid]. Membranes were incubated for 5 min in Ponceau S solution and then washed once with water, photographed, and destained with TBST. Ponceau S staining was used as a Western blotting multiprotein loading control throughout the manuscript instead of a single protein (e.g., β-actin), whose expression might be altered in HF-MSS cells.

Filters were then blocked for 1 h at room temperature with either 5% non-fat dried milk or 5% BSA, and incubated with the primary antibodies overnight at 4 °C. Secondary antibodies α-rabbit and α-mouse were applied the day after for 1 h at room temperature and signal was detected by GE Amersham ECL.

For PCR analysis of XBP1 splicing [35], RNA was extracted from cells by a miRneasy kit (QIAGEN cat. 217004). cDNA was then obtained using a reverse transcription amplification kit (Applied Biosystem) and PCR was run using 54 °C as the compromise annealing temperature for a total of 40 cycles. 10 µL of amplificated products was separated on a 3% agarose gel.

### 4.5. Proliferation Assay

Cell proliferation was monitored using a Click-iT Edu Proliferation assay kit (#C10499, Invitrogen). About 10,000 cells/well were plated in a 96-well plate the day before the experiment, and the manufacturing instructions were followed.

### 4.6. Label-Free Proteomics Analysis

HF-MSS cells were grown to subconfluency, incubated in serum-free medium for 24 h, washed, lysed in proteomic buffer [UREA 6 M, Tris Base 100 mM, CHAPS 2%, Triton X 1%, DTT 50 mM], and treated by the Filter Aided Sample Preparation (FASP) method. Protein concentration was evaluated through Bradford assay (Bio-Rad, Hercules, CA, USA) in order to digest 25 µg of protein by trypsin (Sigma-Aldrich, St. Louis, MO, USA). For protein label-free identification and quantification, tryptic peptides from each sample were analyzed in triplicate with LC-MS/MS using the UltiMateTM 3000 UPLC (Thermo Fisher Scientific, Waltham, MA, USA) chromatographic system coupled to the Orbitrap FusionTM TribridTM (Thermo Fisher Scientific) mass spectrometer. Peptides were loaded on the Trap Cartridge C18 (0.3 mm ID, 5 mm L, 5 μm PS, Thermo Fisher Scientific) and then separated on an EASY-spray AcclaimTM PepMapTM C18 (75 μm ID, 25 cm L, 2 μm PS, Thermo Fisher Scientific) nanoscale chromatographic column. The mobile phase A was 0.1% formic acid in H_2_O and the mobile phase B was 0.1% formic acid in acetonitrile. The flow rate was set at 300 nL/min, with a total run time of 125 min using a chromatographic gradient from 2% to 90% of phase B. Proteomics data were acquired in positive-ion polarity with Data Dependent Acquisition (DDA) mode to trigger precursor isolation and MS2 sequence using N2 as the collision gas for HCD fragmentation. The positive ion voltage was set at 1700 V and the ion transfer tube at 275 °C for desolvation. MS1 scans were performed in the Orbitrap analyzer covering an m/z range of 375–1500 with a resolution of 240,000. A standard automatic gain control (AGC) target and an automatic maximum injection time (MIT) were used. The signal intensity threshold was set to 1.0 × 10^4^ and the MS2 spectra were acquired using a Top Speed method of 3s. In particular, precursor ions with charges of +2 to +5 were used for MS2 sequencing and scanned in the ion trap by setting the following parameters: MS2 isolation window of 1.6 Da, AGC target of 1.0 × 10^4^, dynamic exclusion time of 60 s, and mass tolerance of ±10 ppm were used covering an m/z range of 300–1200. We performed a HCD fragmentation with a fixed collision energy of 30% and a MIT of 70 ms. The mass spectrometry proteomics data were deposited to the ProteomeXchange Consortium via the PRIDE partner repository [37] with the dataset identifier PXD029086.

### 4.7. Proteomics Data Processing

Proteomics raw data were processed using a free computational platform, MaxQuant version 1.6.6.0 (Max-Planck Institute for Biochemistry, Martinsried, Germany). Peak lists, generated in MaxQuant, were searched using Andromeda peptide search engine against the UniProt database (released 2018_04, taxonomy Homo sapiens, 20,874 entries) supplemented with frequently observed contaminants and containing forward and reverse sequences. As reported previously [38,39], carbamidomethylation of cysteines (C) was defined as fixed modification, while oxidation of methionines (M) and deamidation of asparagines (N) and glutamines (Q) were set as variable modifications. The false discovery rate (FDR) both at the protein level and at the peptide level was set at 1%. LFQ Intensity was used to quantify the protein abundance in each sample [40]. Bioinformatics analysis was performed with Perseus version 1.6.10.50 (Max-Planck Institute for Biochemistry, Martinsried, Germany). LFQ intensities were log2 transformed to facilitate the calculation of the protein expression. Site only and reverse and contaminant peptides were removed from the dataset. The minimum number of valid values accepted was set to 2 in at least one group. In this way, we evaluated not only the different protein expression, but also the presence or absence of proteins between the two conditions. Moreover, a univariate statistical analysis was performed with a *p*-value threshold of 0.05 in order to define the significantly different proteins between HF-MSS cells and HF-CT through Volcano Plot visualization (FDR = 0.01 and S0 = 0.05).

Finally, a selection of significantly modulated proteins (*p*-value < 0.05) was loaded into the Ingenuity Pathway Analysis tool (IPA, Qiagen, Hilden, Germany) and subjected to a Core Analysis. IPA maps the modulated proteins on the basis of their functional annotations on Gene Ontology and proprietary databases to identify enriched Canonical Pathways and Disease or Functions. Furthermore, it is possible to infer about the possible upregulation or downregulation of the pathway, disease, or function if the calculated z-score is ≥ 2.00 or ≤ −2.00 respectively.

### 4.8. Electron Microscopy (EM)

Approximately 2 × 10^6^ HF cells cultured in monolayers were prepared for EM as follows. Cells were detached from the dish with trypsin, centrifuged at 180× *g* for 2.5 min, washed 3 times with PBS at 37 °C, fixed with 3.5% glutaraldehyde in 0.1 M sodium cacodylate buffer (NaCaCO) for 1h, and stored at 4 °C. For embedding, cells were post-fixed in 2% OsO_4_ in the same buffer for 2 h and block-stained in saturated uranyl acetate replacement. After dehydration, specimens were embedded in epoxy resin (Epon 812). Ultrathin sections were cut using a Leica Ultracut R microtome (Leica Microsystem, Austria) with a Diatome knife (DiatomeLtd. CH-2501 Biel, Switzerland) and double-stained with uranyl acetate replacement and lead citrate. Sections were viewed and photographed in a Morgagni Series 268D electron microscope (FEI Company, Brno, Czech Republic) equipped with a Megaview III digital camera and Analy-SIS software (Olympus Soft Imaging Solutions).

Quantitative analysis was done on random micrographs of HF cell cultures (CT and MSS). The percentage of cells with cytoplasmic accumulation of vacuoles delimited by membranes and containing electron-dense material was determined in micrographs taken at either 5600 or 7100 of magnification (sample size: 100 cells/group).

## Figures and Tables

**Table 1:** Selected biological processes altered in HF-MSS, based on Gene Ontology enrichment analysis.

Term ID ^1^	Term Description ^2^	Observed Gene Count ^3^	Background Gene Count ^4^	False Discovery Rate ^5^
GO:0016071	mRNA metabolic process	83	667	2.5 × 10^−21^
GO:0000398	mRNA splicing, via spliceosome	45	284	6.16 × 10^−15^
GO:0006397	mRNA processing	54	456	2.70 × 10^−13^
GO:1903311	regulation of mRNA metabolic process	36	238	1.89 × 10^−11^
GO:0043488	regulation of mRNA stability	21	113	4.54 × 10^−8^
GO:0050684	regulation of mRNA processing	18	114	4.33 × 10^−6^
GO:0048024	regulation of mRNA splicing, via spliceosome	15	77	4.38 × 10^−6^
GO:0006413	translational initiation	30	142	1.17 × 10^−12^
GO:0006412	translation	44	362	3.99 × 10^−11^
GO:0006417	regulation of translation	38	327	3.73 × 10^−9^
GO:0002181	cytoplasmic translation	15	57	1.90 × 10^−7^
GO:0006446	regulation of translational initiation	8	71	0.0246
GO:0006614	SRP-dependent cotranslational protein targeting to membrane	23	92	6.11 × 10^−11^
GO:0016192	vesicle-mediated transport	139	1699	6.02 × 10^−21^
GO:0060627	regulation of vesicle-mediated transport	30	480	0.0077
GO:0006892	post-Golgi vesicle-mediated transport	9	83	0.0197
GO:0055114	oxidation-reduction process	94	932	4.04 × 10^−9^
GO:0015980	energy derivation by oxidation of organic compounds	17	217	0.0112
GO:0044281	small molecule metabolic process	134	1779	1.21 × 10^−17^
GO:0062012	regulation of small molecule metabolic process	21	332	0.0272

^1^ Unique seven-digit identifier prefixed by GO. ^2^ Textual description of the term. ^3^ Number of genes linked to that GO term in the input list. ^4^ Number of genes linked to a GO term in the entire background set. ^5^ Expected ratio of the number of false positive classifications (false discoveries) to the total number of positive classifications.

**Table 2:** Selected molecular functions altered in HF-MSS, based on Gene Ontology enrichment analysis.

Term ID ^1^	Term Description ^2^	Observed Gene Count ^3^	Background Gene Count ^4^	False Discovery Rate ^5^
GO:0003723	RNA binding	86	850	2.57 × 10^−17^
GO:0003729	mRNA binding	36	198	2.76 × 10^−13^
GO:0003727	single-stranded RNA binding	13	80	0.00011
GO:0003725	double-stranded RNA binding	11	70	0.00058
GO:0019843	rRNA binding	10	60	0.00079
GO:0008135	translation factor activity, RNA binding	11	84	0.0021
GO:0000049	tRNA binding	8	56	0.0079
GO:0061980	regulatory RNA binding	5	34	0.0424
GO:0016491	oxidoreductase activity	75	716	8.70 × 10^−16^
GO:0016860	intramolecular oxidoreductase activity	12	47	5.01 × 10^−6^
GO:0000166	nucleotide binding	134	2097	2.45 × 10^−12^
GO:0017076	purine nucleotide binding	108	1865	1.36 × 10^−7^
GO:0030554	adenyl nucleotide binding	79	1524	0.00039
GO:0008092	cytoskeletal protein binding	71	882	4.82 × 10^−10^
GO:0046961	proton-transporting ATPase activity, rotational mechanism	9	21	4.97 × 10^−6^
GO:0008553	proton-exporting ATPase activity, phosphorylative mechanism	9	23	8.55 × 10^−6^
GO:0015078	proton transmembrane transporter activity	12	120	0.0082
GO:0005178	integrin binding	19	122	2.30 × 10^−6^
GO:0050839	cell adhesion molecule binding	24	200	3.68 × 10^−6^
GO:0098634	cell-matrix adhesion mediator activity	3	7	0.0229

^1^ Unique seven-digit identifier prefixed by GO. ^2^ Textual description of the term. ^3^ Number of genes linked to that GO term in the input list. ^4^ Number of genes linked to a GO term in the entire background set. ^5^ Expected ratio of the number of false positive classifications (false discoveries) to the total number of positive classifications.

**Table 3:** Selected cellular components altered in HF-MSS, based on Gene Ontology enrichment analysis.

Term ID ^1^	Term Description ^2^	Observed Gene Count ^3^	Background Gene Count ^4^	False Discovery Rate ^5^
GO:0043226	organelle	566	12432	1.37 × 10^−50^
GO:0043229	intracellular organelle	560	12193	3.30 × 10^−50^
GO:0043227	membrane-bounded organelle	516	11244	1.07 × 10^−37^
GO:0005783	endoplasmic reticulum	120	1796	9.40 × 10^−13^
GO:0005788	endoplasmic reticulum lumen	30	299	1.86 × 10^−6^
GO:0005793	endoplasmic reticulum-Golgi intermediate compartment	13	113	0.00095
GO:0031982	vesicle	173	2318	4.41 × 10^−24^
GO:0031410	cytoplasmic vesicle	166	2226	6.49 × 10^−23^
GO:0099503	secretory vesicle	96	948	1.62 × 10^−20^
GO:0030139	endocytic vesicle	35	275	8.75 × 10^−10^
GO:0045335	phagocytic vesicle	23	122	2.25 × 10^−9^
GO:0042470	melanosome	35	105	1.55 × 10^−20^
GO:1990904	ribonucleoprotein complex	89	770	2.33 × 10^−22^
GO:0030532	small nuclear ribonucleoprotein complex	11	67	0.00021
GO:0005773	vacuole	74	682	4.05 × 10^−17^
GO:0005775	vacuolar lumen	30	172	2.47 × 10^−11^
GO:0005774	vacuolar membrane	31	311	1.36 × 10^−6^
GO:0005764	lysosome	67	582	1.24 × 10^−16^
GO:0015629	actin cytoskeleton	42	432	1.75 × 10^−8^
GO:0032432	actin filament bundle	12	57	1.38 × 10^−5^

^1^ Unique seven-digit identifier prefixed by GO. ^2^ Textual description of the term. ^3^ Number of genes linked to that GO term in the input list. ^4^ Number of genes linked to a GO term in the entire background set. ^5^ Expected ratio of the number of false positive classifications (false discoveries) to the total number of positive classifications.

**Table 4:** Selected altered pathways in HF-MSS, based on KEGG enrichment analysis.

Term ID ^1^	Term Description ^2^	Observed Gene Count ^3^	Background Gene Count ^4^	False Discovery Rate ^5^
hsa03040	spliceosome	26	130	3.14 × 10^−10^
hsa03010	ribosome	21	130	3.48 × 10^−7^
hsa03013	RNA transport	14	159	0.0074
hsa04145	phagosome	26	145	1.35 × 10^−9^
hsa04142	lysosome	24	123	1.39 × 10^−9^
hsa01100	metabolic pathways	99	1250	1.52 × 10^−13^
hsa00020	citrate cycle (TCA cycle)	9	30	7.00 × 10^−5^
hsa01212	fatty acid metabolism	10	48	0.00024
hsa01230	biosynthesis of amino acids	11	72	0.00075
hsa00010	glycolysis/Gluconeogenesis	10	68	0.0017
hsa00030	pentose phosphate pathway	5	30	0.0259

^1^ Unique identifier for each KEGG object which takes the form of a prefix followed a five-digit number. ^2^ Textual description of the term. ^3^ Number of genes linked to that KEGG term in the input list. ^4^ Number of genes linked to a KEGG term in the entire background set. ^5^ Expected ratio of the number of false positive classifications (false discoveries) to the total number of positive classifications.

**Table 5:** Selected canonical pathways altered in HF-MSS, based on Ingenuity Pathway Analysis.

Ingenuity Canonical Pathway ^1^	−log (*p*-Value) ^2^	z-Score ^3^
phagosome maturation	14.6	
EIF2 signaling	14	−2.357
caveolar-mediated endocytosis signaling	7.97	
fatty acid β-oxidation I	7.88	2.53
TCA cycle II (Eukaryotic)	6.89	2.121
isoleucine degradation I	6.83	2.236
mitochondrial dysfunction	6.78	
valine degradation I	4.99	2.236
tryptophan degradation III (Eukaryotic)	4.49	1.342
spliceosomal cycle	3.51	−2.646
tRNA charging	2.43	−1.342

^1^ Textual description of the term. ^2^ Negative logarithm of *p*-value. ^3^ Also called standard score, is the number of standard deviations by which the value of a raw score is above or below the mean value of what is being observed or measured.

**Table 6:** Selected disease or functions that are predicted to be increased or decreased on the basis of differential proteins in HF-MSS, based on Ingenuity Pathway Analysis.

Disease or Functions Annotation ^1^	*p*-Value	z-Score ^2^	Predicted Activation State ^3^
fatty acid metabolism	4.57 × 10^−12^	2.017	Increased
cellular homeostasis	5.29 × 10^−11^	2.129	Increased
cell death of tumor cell lines	8.41 × 10^−24^	2.15	Increased
endocytosis by eukaryotic cells	5.24 × 10^−11^	2.311	Increased
metabolism of glycosphingolipid	0.00000134	2.387	Increased
cell death of tumor cells	1.13 × 10^−13^	2.416	Increased
sensitivity of cells	0.000000386	2.56	Increased
phagocytosis	3.93 × 10^−8^	2.708	Increased
phagocytosis of cells	0.000000438	2.714	Increased
degradation of extracellular matrix	7.31 × 10^−8^	2.905	Increased
cell death of cancer cells	1.17 × 10^−10^	2.995	Increased
cell death of osteosarcoma cells	1.76 × 10^−12^	3.545	Increased
homologous recombination	9.48 × 10^−9^	−3.637	Decreased
accumulation of lipid	2.49 × 10^−9^	−3.636	Decreased
homologous recombination of cells	5.85 × 10^−9^	−3.499	Decreased
glucose metabolism disorder	0.00000102	−3.279	Decreased
DNA recombination	6.51 × 10^−9^	−3.235	Decreased
motor dysfunction or movement disorder	1.01 × 10^−18^	−2.934	Decreased
movement Disorders	9.11 × 10^−19^	−2.666	Decreased
lysosomal storage disease	2.68 × 10^−10^	−2.591	Decreased
repair of DNA	0.000000166	−2.545	Decreased
cell death of breast cancer cell lines	9.12 × 10^−8^	−2.374	Decreased
astrocytosis	0.00000147	−2.219	Decreased
degeneration of central nervous system	8.66 × 10^−9^	−2.218	Decreased
neurodegeneration	0.000000465	−2.185	Decreased
degeneration of nervous system	0.000000827	−2.128	Decreased
amyloidosis	7.3 × 10^−13^	−2.08	Decreased
degeneration of brain	1.46 × 10^−8^	−2.032	Decreased

^1^ Textual description of the term. ^2^ Also called standard score, is the number of standard deviations by which the value of a raw score is above or below the mean value of what is being observed or measured. ^3^ Prediction based on the observed gene expression changes in the dataset.

## Data Availability

Data available upon request.

## Supplementary Materials

The following are available online at https://www.mdpi.com/article/10.3390/ijms222212449/s1. Figure S1. The viability and amount of apoptotic cell death of HF-CT and HF-MSS cells are comparable. Figure S2. Reproducibility of the proteomic analysis. Figure S3. HF-MSS proliferation assay. Table S1. Proteomic. Table S2. Biological Processes. Table S3. Molecular Functions. Table S4. Cellular Components. Table S5. KEGG Pathways. Table S6. Canonical Pathways. Table S7. Disease or functions.
