# Supplementary figures and images for: Proteomic Analysis of Marinesco–Sjogren Syndrome Fibroblasts Indicates Pro-Survival Metabolic Adaptation to SIL1 Loss
Source: Int J Mol Sci. 2021 Nov 18;22(22):12449. doi: 10.3390/ijms222212449 (PMC8620507; doi:10.3390/ijms222212449)

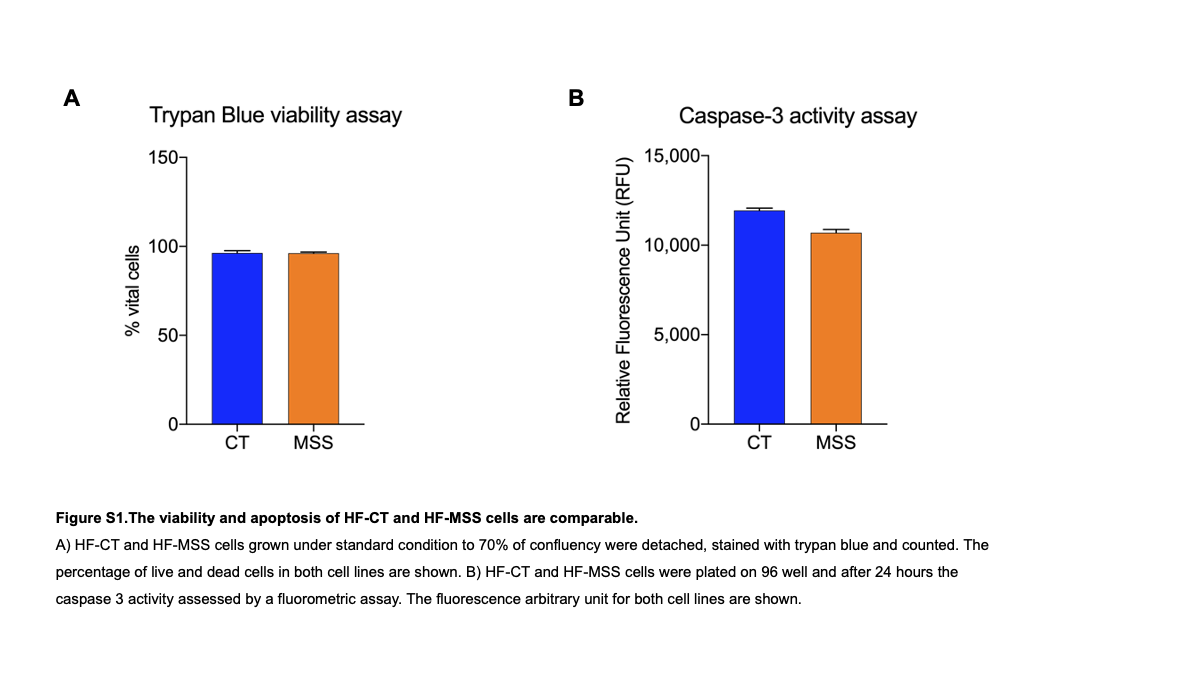

Supplement: Supplementary file 1 [file ijms-22-12449-s001.zip › Supplementary figures_Proof/Diapositiva1.tiff]

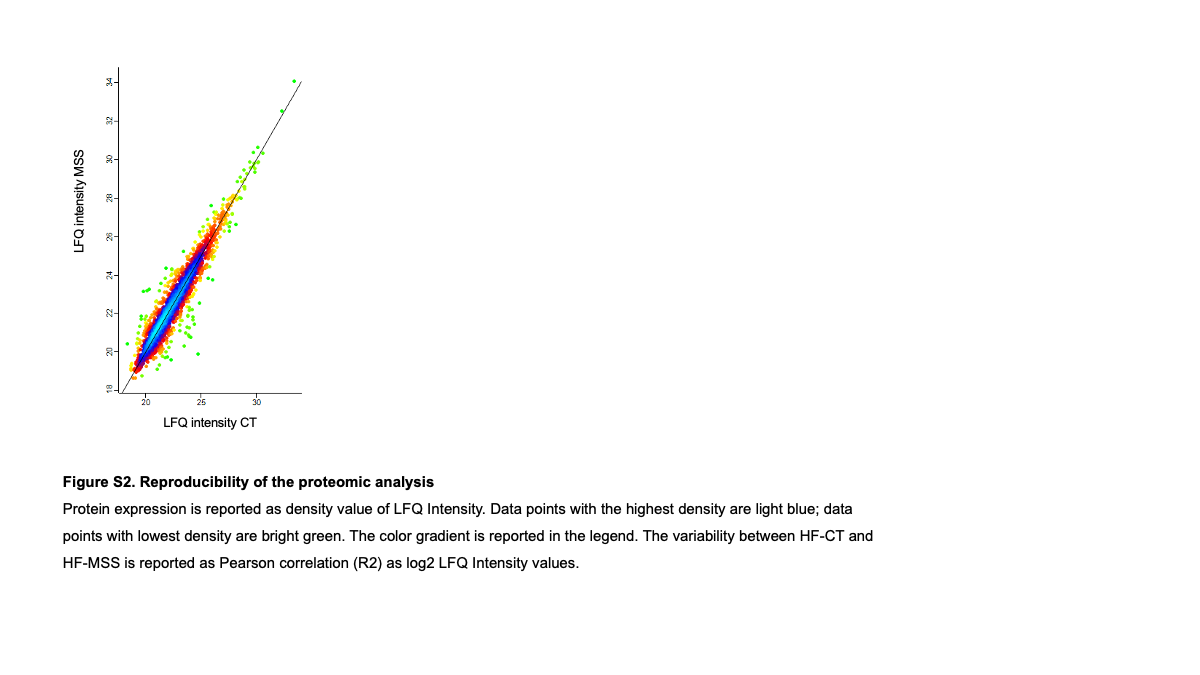

Supplement: Supplementary file 1 [file ijms-22-12449-s001.zip › Supplementary figures_Proof/Diapositiva2.tiff]

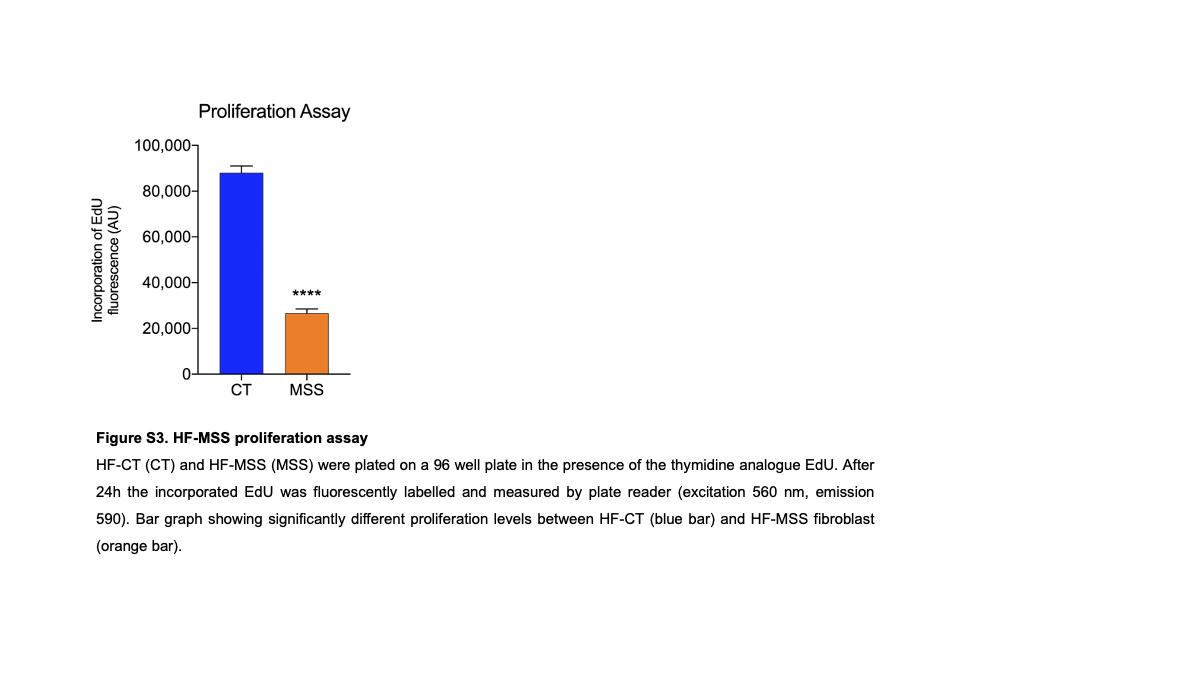

Supplement: Supplementary file 1 [file ijms-22-12449-s001.zip › Supplementary figures_Proof/Diapositiva3.tiff]
